# Editorial: Reviews in neurorehabilitation

**DOI:** 10.3389/fneur.2024.1366899

**Published:** 2024-02-13

**Authors:** Teresa Paolucci, Won-Seok Kim, Pierluigi Zoccolotti

**Affiliations:** ^1^Department of Oral Medical Sciences and Biotechnology, S. Spirito Hospital of Pescara, University of G. D'Annunzio of Chieti and Pescara, Physical and Rehabilitation Medicine, Chieti, Italy; ^2^Department of Rehabilitation Medicine, Seoul National University College of Medicine, Seoul National University Bundang Hospital, Seongnam, Republic of Korea; ^3^Department of Psychology, Sapienza University of Rome, Rome, Italy; ^4^Tuscany Rehabilitation Clinic, Montevarchi, Italy

**Keywords:** neurorehabilitation, telerehabilitation, motor function, cognitive processes, dysphagia, mental health, robotic systems, respiratory difficulties

## Introduction

The Research Topic on “*Reviews in neurorehabilitation*” has collected several systematic and scoping reviews and meta-analyses about relevant rehabilitation topics. The emerging picture underscores the complexity of the issues connected with caring for patients with neurological disabilities of all ages and places. Rehabilitative care in neurological disorders presupposes an individual rehabilitation plan based on the alliance of a multidisciplinary team aimed at recovering function by combining traditional techniques with the most innovative and novel models of robotic rehabilitation and virtual reality.

## Functional motor recovery

Traditionally, several rehabilitative interventions are connected to different aspects of motor function. In the Research Topic, two articles analyse the recovery post-stroke. Specifically, Khan et al. review the effects of various forms of functional electrical stimulation on upper limb in post-stroke recovery. This evidence may be instrumental in identifying areas for further research with this promising paradigm. It is commonly observed that lower limbs recover faster than upper limbs. Abdullahi et al. present a theoretical analysis of the possible reasons for such asymmetry. The authors posit that it is the more frequent use of the lower limb to carry out this asymmetry. Based on this interpretation, upper limb rehabilitation may be aided by paradigms which allow highly repetitive practice, such as robotic rehabilitation, Wii gaming, and constraint-induced movement therapy.

Other reviews are concerned with various neurological conditions. Faccioli, Pagliano et al. focus on the management and motor rehabilitation of cerebral palsy in children and adolescents. Evidence has accumulated over the years, indicating the importance of setting updated, evidence-based guidelines for clinical practice. The authors underscore the importance of identifying individual goals and interventions within a multidisciplinary, family-centered, evidence-based management. Considering the young age of patients, it is advised to use motor rehabilitation programs which actively engage the subject, preferably through intensive and time-limited practice. Faccioli, Cavalagli et al. systematically review the evidence on the gait analysis patterns and rehabilitative intervention in hereditary spastic paraplegia and indicate some avenues of possible interventions.

A critical aspect of rehabilitation is the long-term effect on the patient's wellbeing. In their scoping review, Paolucci et al. examine the various options available for stroke patients in the chronic stage and underscore the importance of pursuing rehabilitation goals and not limiting them to maintenance. Indeed, the analysis of the relevant literature supports the efficacy of rehabilitative treatments over motor function following rehabilitation in the chronic stage with improvements in the quality of life.

The element that emerges from these various reviews is the need to have rehabilitation protocols that are more shared in terms of duration and intensity in the neurorehabilitation paths, both in the acute and chronic phases of the post-stroke patient.

A relevant point for patient recovery and successful rehabilitation is the association of conventional rehabilitation techniques with innovative systems, especially in rehabilitation training. Wu et al. illustrate the use of the Lokomat^®^ robotic-orthosis system in patients with stroke and examine this new paradigm compared to conventional physical therapy for gait training and balance. Similarly, Cheng et al.'s scoping review focuses on the putative neuroplastic mechanisms of transcranial magnetic stimulation (rTMS) in stroke rehabilitation path. Their analysis underscores the presence of excitation and synchronization of neural activity as well as changes in functional connectivity between brain regions as assessed by functional magnetic resonance imaging (fMRI). TMS represents a supportive therapy to increase the recovery of residual motor function of the upper and lower limbs in the post-stroke patient.

In the context of instrumental physical therapy, the systematic review and meta-analysis by Wang et al. analyses the effectiveness of neuromuscular electrical stimulation (NMES) in the treatment of post-stroke dysphagia. NMES appears to have positive effects when combined with standard therapy on the quality of life, reducing the incidence of complications and promoting the recovery of swallowing function. However, its safety needs to be further confirmed.

Overall, the last years have seen an impressive growth of technologies generating innovative interventions for selective problems due to neurological conditions. The accumulation of evidence allows us to draw some conclusions on the relative efficacy of the various techniques and paradigms.

## Telerehabilitation and computer-assisted rehabilitation

Telerehabilitation approaches could represent a novel resource supporting traditional rehabilitative paths because they make rehabilitation usable even at the patient's home, including the caregiver, throughout the treatment process and in the neurological chronic phase. In their mini-review, Xing et al. illustrate the characteristics of information and communication technologies (ICTs) that are most relevant for stroke interventions and discuss which types of training could be effectively offered with this paradigm. Overall, there is more and more evidence that telerehabilitation is not generally inferior to classical therapies carried out in the clinical setting when doses and intensities are comparable.

A relevant target of telerehabilitation is intervening in cognitive disturbances, a crucial sequela of stroke and other neurological conditions. Maggio et al. present an expert review highlighting software and devices currently available for training cognitive processes at home. Vuori et al. carry out a systematic review of the pertinent literature. In general, adherence and patient satisfaction are positive aspects of these interventions; by contrast, evidence supporting the usefulness of these trainings is still insufficient and based on studies with low methodological quality. Despite these limitations, the perspective opened by telerehabilitation approaches is promising and more high-quality studies are urgently needed.

A potentially promising area of use of telerehabilitation concerns mental health. While there is considerable evidence on the effectiveness of this paradigm on depression and anxiety in adults, less is known in the case of patients with neurological disorders. Zhang et al. systematically review this recent literature, underscoring the effectiveness of web-based interventions on depression and anxiety symptoms in patients with neurological disorders. They note that different variables, including intervention type and duration, modulate the efficacy of these interventions.

Telerehabilitation represents a key avenue for developing interventions for neurological conditions. It appears that more high-quality evidence needs to be acquired to reach definite conclusions on the effectiveness of the various therapeutic programs. However, even the current limited knowledge underscores the far-reaching potentiality of this approach in various areas of cognitive and mental disturbances, notwithstanding the possibility of selective use also for motor deficits. Importantly, in the ever-increasing need for neurorehabilitation interventions closely associated with an aging population, telerehabilitation may favor the expansion of rehabilitation services by significantly reducing costs and reaching patients in remote areas.

## Additional therapeutic interventions for cognitive problems

One relevant area in cognitive disturbances concerns the interventions aimed at reducing the incidence of perioperative neurocognitive disorders. In a systematic review and meta-analysis of the relevant literature, Zhao et al. examine the effect of these interventions on postoperative delirium as well as on possible decline in cognitive performances.

In the field of complementary and alternative therapies (CAM-Therapy), Liu et al. analyse the efficacy and safety of multiple acupuncture therapies for post-stroke cognitive impairment, specifically ophthalmic acupuncture plus cognitive training. Due to the low methodological quality of the included studies, the findings need to be treated with caution. However, there were no reports of serious adverse effects, and acupuncture treatment appears safe and reliable.

## Sensory-perceptual rehabilitation

Two reviews examine interventions for selective sensory-perceptual problems following neurological conditions. The study by Xie et al. examines the respiratory difficulties and mortality following severe cervical spinal cord injury (CSCI) as a primary consequence of malfunctions of respiratory pathways and the paralyzed diaphragm but also tries to respond to the question of whether there are other potential therapeutic targets to consider. The authors propose that magnetic resonance neuroimaging holds promise in examining respiratory function post-CSCI, studying respiratory plasticity in the brain and spinal cord to guide future clinical work.

Chen et al. present a meta-analysis of the effects of vestibular rehabilitation training (VRT) combined with anti-vertigo drugs on vertigo and balance function in patients with vestibular neuronitis. Evidence generally favors the use of this combined therapy to alleviate vestibular dysfunction, such as vertigo and vomiting, improving the quality of life of affected patients. The paucity of adverse reactions is another positive asset of this type of intervention.

## Final remarks

In conclusion, some emerging properties arise from an overview of the reviews and meta-analyses of the Research Topic. First, the accumulating evidence on different rehabilitative interventions fosters the possibility of developing individualized, patient-centered management of patients with neurological disorders; this may entail engaging the patient in goal-directed, skill-based rehabilitative interventions suitable for the needs and preferences of the person and their family. Second, the complexity and articulation of available techniques call for a multidisciplinary and interdisciplinary rehabilitative teams. Third, several technological advancements bloomed in the last years, opening new frontiers in neurorehabilitation. The reviewed evidence points to the efficacy of some of these techniques in enlarging the portfolio of rehabilitators. It appears that combining traditional rehabilitative techniques with new innovative technologies opens an ever more promising perspective in neurorehabilitation. Fourth, one area connected with technological advancements is that of telerehabilitation. We are only starting to see the potentialities of this perspective. Of particular interest is the possibility of broadening the scope of rehabilitation services that are offered to patients with neurological syndromes and their families.

While technological advances are fundamental to increasing the effectiveness and pervasiveness of rehabilitation techniques, it is important to stress that, however elaborate, technologies do not abolish the need for accurate functional diagnosis that remains the essential basis for optimally setting up a person-centered rehabilitative project. Furthermore, we still need to better understand the individual characteristics of those who most likely will respond to one intervention versus another.

As Guest Editors for this Research Topic, we hope that you enjoy these extremely interesting manuscripts.

## Author contributions

TP: Writing—original draft. W-SK: Writing—review & editing. PZ: Writing—review & editing.

